# 

**Published:** 1892-04

**Authors:** 


### CONTENTS.

COMMUNICATIONS.

The Work and Duties of the Ohio State Dental Society ................ 151
The Skin and its Appendages, with Special Reference to the Development of the Teeth ................ 154
Pental, the New Anæsthetic ................ 161
Decolorization of the Teeth ................ 168
Permanent Closure of the Jaws, with Report of a Case ................ 170
Surgery of the Antrum ................ 178
A New Oxyphospate for Crown Setting ................ 184
President's Address ................ 188
The World's Columbian Dental Congress ................ 192

EDITORIAL.

The Seventh District Dental Society of Ohio ................ 201
Died ................ 202

